# Case Report: Whipple’s disease pneumonia caused by inhalation of gas or liquid

**DOI:** 10.3389/fmed.2025.1664867

**Published:** 2025-10-09

**Authors:** Xiaokui Sun, Huizhi Zhu

**Affiliations:** ^1^The First Clinical Medical College of Anhui University of Chinese Medicine, Hefei, China; ^2^The First Affiliated Hospital of Anhui University of Chinese Medicine, Hefei, China

**Keywords:** aspiration pneumonia, *Tropheryma whipplei* (TW) infection, aflatus, meropenem, ceftriaxone, sulfamethoxazole/trimethoprim (SMZ)

## Abstract

*Tropheryma whipplei* (*T. whipplei*) *i*s a Gram-positive bacterium that causes rare clinical infections, which primarily manifest as gastrointestinal infections and, more rarely, pulmonary diseases. Furthermore, immunosuppression is considered a predominant risk factor for *T. whipplei* infection. Several studies have indicated an association between *T. whipplei* infection and immunocompromised conditions; however, a few studies have also revealed that aspiration may be a potential predisposing factor for *T. whipplei-*related aspiration pneumonia. This case study reports two different cases of aspiration-induced *T. whipplei* pneumonia, each exhibiting unique clinical manifestations, imaging features, and therapeutic responses. Both patients had a history of aspiration, and their diagnoses were confirmed via mNGS. They were treated with sulfamethoxazole–trimethoprim (SMZ), which effectively alleviated the symptoms of mild *T. whipplei* pneumonia. However, in one case, the patient suffered from a severe co-infection of *T. whipplei* and *Aspergillus* following a drowning incident, which was effectively treated with meropenem and voriconazole co-therapy. This study provides novel data on *T. whipplei* infection and improves clinical awareness of aspiration-related pneumonia.

## 1 Introduction

*Tropheryma whipplei* (*T. whipplei*) is a rare Gram-positive bacterium and a member of the Actinomycetes class (1). *Tropheryma whipplei* has not been frequently documented in the clinical literature due to challenges in culturing, unusual clinical symptoms and imaging characteristics, and diagnostic limitations. However, with the advancements in metagenomic next-generation sequencing (mNGS) technology, more cases of *T. whipplei* infection are being identified. The literature suggests that immunodeficiency is a predominant underlying factor in *T. whipplei* infections. Only a few external factors, such as environmental inhalation, have been suggested; therefore, the bacterium is frequently not regarded as a typical clinical pathogen (2). Pulmonary *T. whipplei* infections have been predominantly observed in immunosuppressed populations, with few definitive cases attributed to aspiration. Clinically, aspiration is a known cause of pneumonia. Although gases and liquids are frequently aspirated, *T. whipplei* is often underrecognized. The radiographic manifestations and therapeutic approaches associated with the aspiration of gases and liquids may differ significantly. This study reports two *T. whipplei* pneumonia cases caused by aspiration, one associated with gas aspiration and the other resulting from sewage aspiration. Both patients were diagnosed using mNGS and treated with individualized management strategies based on the previous studies, leading to favorable therapeutic outcomes.

### 1.1 Case presentation 1: *TW pneumonia* induced by gas aspiration

**Figure 1:**
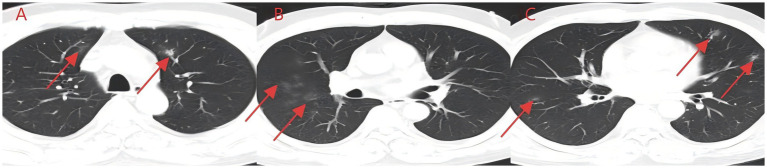
**(A–C)** Aspiration of irritating gas chest CT (case 1). Asymmetric scattered ground-glass shadows were seen in both lungs, with no obvious solid shadows or pleural effusion.

A middle-aged Asian man with a 2-day history of cough and expectoration was referred from the outpatient department to the Department of Respiratory Medicine. His symptoms started after exposure to chemical fumes at night and included a cough with expectoration secondary to pharyngeal irritation, as well as acid reflux and nausea. Due to the transient nature of the incident, it was not feasible to collect and analyze the irritating gas at the time. However, based on the patient’s description, it was inferred that they had inhaled substances similar to formaldehyde. Symptoms were more pronounced before bedtime and in the morning. The patient denied other systemic discomfort, and physical examination indicated no significant abnormalities. Standard laboratory tests were performed at admission, including a complete blood count, urinalysis, stool analysis, coagulation profile, tumor markers, respiratory virus screening, fungal (1–3)-*β*-D-glucan testing, and a *Cryptococcus neoformans* capsular antigen assay; none of these tests produced any significant findings. An external chest CT scan demonstrated bilateral ground-glass opacities (Figure 1). Due to a previous medical history of hypertension, at the time of admission, the patient was treated with irbesartan and oral hydrochlorothiazide. He denied having any additional long-term medical conditions or recognized food or drug allergies. To prevent potential bacterial infections, empirical antimicrobial therapy was started with 0.4 g of intravenous (IV) moxifloxacin once a day (qd). After ruling out any contraindications, an electronic bronchoscopy was performed, and alveolar lavage fluid samples were subjected to mNGS analysis. Bronchoscopic findings were normal, whereas mNGS identified *T. whipplei* with a sequence count of 372, and no other pathogenic microorganisms were detected. Therefore, the antimicrobial regimen was modified to IV ceftriaxone (CTX) 2 g/day in combination with oral sulfamethoxazole–trimethoprim (SMZ) twice daily for targeted infection control. After 6 days of treatment, the patient reported substantial improvement in cough and expectoration. However, despite medical advice, he refused a follow-up chest CT scan and additional inpatient care, opting to be discharged voluntarily. The patient showed no symptoms during the next phone follow-up.

### 1.2 Case presentation 2: *TW* mixed aflatoxin pneumonia induced by the aspiration of sewage

**Figure 2:**
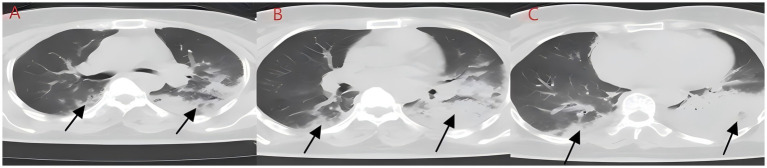
**(A–C)** Chest CT after aspiration of sewage (case 2). Diffuse infection can be seen in both lungs, mainly in the lower lobe of the left lung, with obvious bronchial inflation signs.

**Figure 3:**
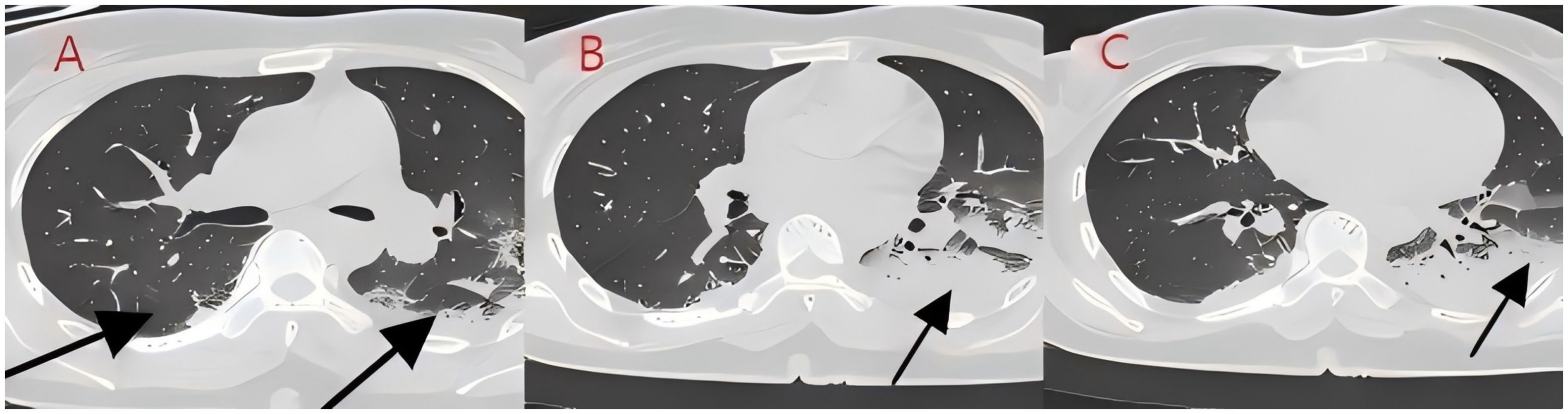
**(A–C)** Chest CT after 2 days of treatment (case 2). After bronchoscopy and antibiotic treatment for bilateral lung infections, the infection foci were absorbed significantly.

**Figure 4:**
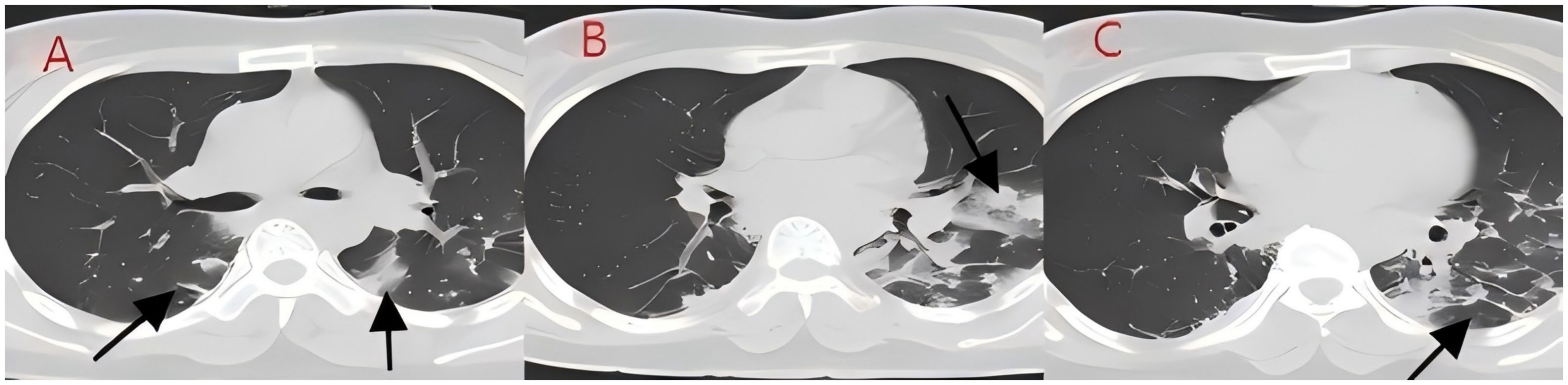
**(A–C)** Chest CT after 8 days of treatment (case 2). The infection in both lungs has significantly absorbed, large areas of consolidation shadows have been absorbed, and only some cord-like shadows can be seen.

**Figure 5:**
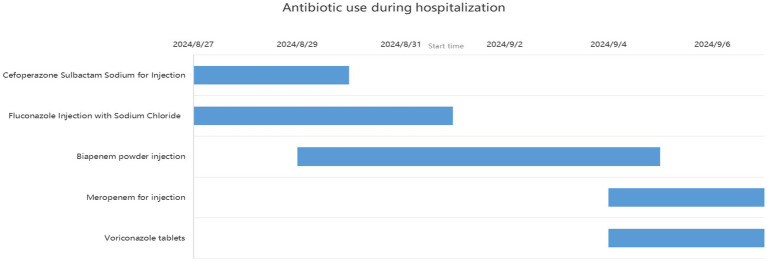
Records of antibiotic use during hospitalization (case 2).

We present another case of a middle-aged Asian woman who was admitted to the hospital after drowning and inhaling a large amount of sewage. The patient inhaled stagnant wastewater from domestic sewage discharge. She showed noticeable chills and vomited a black, muddy substance while being transported. Upon admission, head, chest, and belly CT scans, along with related blood testing, were performed. Due to her critical condition, she was transferred to the ICU. Physical examination at admission revealed that her body temperature was 36.3 °C, pulse was 81 beats/min, respiration rate was 16 breaths/min, and blood pressure was 112/61 mmHg. The patient was conscious but had signs of fatigue and significant chills. Her scalp skin had a 3 × 1 cm tear. Her cardiac examination was normal; however, lung auscultation revealed bilateral wet rales, which were more pronounced in the left lung area. Furthermore, she had normal limb muscle strength and tone. There are no signs of meningeal irritation. The patient denied a history of underlying medical conditions or food or drug allergies. Emergency CT scan showed subarachnoid hemorrhage, and infectious lesions were observed in both lungs, with the left lung being primarily affected (Figure 2). The left lung also exhibits patchy, hyperdense shadows and a little pleural effusion. Moreover, her potassium levels were 2.96 mmol/L, and osmotic pressure was 288 mOsm/Kg. Similarly, the coagulation characteristics analysis revealed a D-dimer level of 3,990 μg/L. Arterial blood gas analysis showed her lactate level to be 2.50 mmol/L, pH (T) to be 7.37, pCO_2_ (T) to be 40.60 mmHg, and pO_2_ (T) to be 55.60 mmHg. Her complete blood count and C-reactive protein test results revealed that her white blood cell count was 19.77 × 10^9^/L, whereas the C-reactive protein level was 0.51 mg/L. At admission, she was diagnosed with traumatic subarachnoid hematoma, aspiration pneumonia, type I respiratory failure, and drowning. After she was admitted to the ICU, she was given anti-infective drugs: cefoperazone–sulbactam (3.0 IV via VP every 8 h) combined with fluconazole (FLZ) injection [0.4 g via IV drip (IV gtt) once daily] to fight infection. After 2 days, the patient was re-examined due to stable vital signs, and chest CT revealed that lung infections had improved compared to the initial findings (Figure 3). After consultation with a clinical pharmacist, cefoperazone–sulbactam was changed to biapenem (0.3 g IV gtt qd every 8 h) combined with FLZ (0.4 g IV gtt qd) to remove the microorganisms. After 5 days, due to recurrent fever, she was transferred to the respiratory department and her anti-infection regimen was adjusted to oral therapy: meropenem (Mpm; 1 g IV every 8 h) combined with voriconazole tablets (400 mg every 12 h). After ruling out contraindications, tracheoscopy was performed on LB2 and LB3, with brushing and BAL of the left upper lobe. Furthermore, cytology, acid-fast stain, cell differential count, culture, and mNGS analysis of the lavage fluid were performed. Both lungs indicated improved infection lesions in the chest re-examination (Figure 4). The patient was discharged after 12 days of admission due to her inability to pay hospitalization expenses. The lavage fluid mNGS revealed the presence of *T. whipplei* (sequence number: 4683) and *Aspergillus flavus* (sequence number 9). During hospitalization, the primary medication was a dimorphic dose, but only the last dose was consistent with the mNGS report (Figure 5).

## 2 Discussion

*Tropheryma whipplei* was first reported in 1907 and taxonomically classified as a Gram-positive bacterium that belonged to the Radiobacteria/Actinomycetes class. It is widely distributed in soil and sewage (1, 2). Clinically, *T. whipplei* has been found to predominantly colonize the human digestive and respiratory systems; however, pulmonary infections are relatively rare. *Tropheryma whipplei* pneumonia is most commonly observed in immunocompromised patients, such as those with HIV, cancer, or those under prolonged use of oral immunosuppressants. This study reports on two patients who lacked such histories but had clear records of aspiration events. Aspiration denotes the inhalation of substances, such as oral or stomach contents, sewage, gasoline, kerosene, or other pollutants into the respiratory tract, resulting in aspiration pneumonia and possible lung injury. The disease etiology in the two cases was different. In case 1, the patient experienced a dry cough with acid reflux immediately after inhaling an irritating gas before symptom onset, indicating the specific manifestation of dominant aspiration. The significant clinical signs included audible and prominent rales in both lungs, and pulmonary imaging revealed scattered ground-glass opacities. In case 2, the patient inadvertently inhaled a substantial quantity of sewage, leading to the onset of a cough accompanied by black, mud-like expectoration, vomiting, fever, and chills. Imaging revealed significant consolidation in both lungs, and the infection markers were significantly elevated.

*T. whipplei* bacteria are commonly found in the saliva of healthy individuals (3). Several studies have indicated favorable detection rates of 19 and 35% in saliva samples from normal subjects (4, 5), suggesting that *T. whipplei* may cause aspiration pneumonia. These patients met the diagnostic criteria for aspiration pneumonia based on high-risk factors, symptoms, signs, and pulmonary imaging findings (6). Moreover, since *T. whipplei* colonizes the digestive system, 12-proctoscopy biopsy with PAS staining is used as the gold standard for diagnosis (7, 8). In this study, the patients experienced acid reflux, nausea, and vomiting at disease onset, potentially aspirating gastric contents containing *T. whipplei*. Gastroenteroscopy was not conducted during hospitalization due to inadequate diagnostic experience and insufficient patient compliance. In case 2, the patient inadvertently swallowed river water near their residence, which contained domestic sewage discharge and potentially harbored *T. whipplei.* Drowning is a prevalent cause of accidental mortality; however, aspiration pneumonia following drowning is clinically rare, and there are limited large-scale clinical data and research available. Fei Guang Yu *et al.* analyzed 18 cases of aspiration pneumonia and revealed flocculent high-density and patchy shadows as the principal imaging alterations, as well as pathogenic bacteria and mixed fungus infections in sputum cultures (9). Yang Nan cultured sputum samples of 156 drowning children and found a higher detection rate of *T. whipplei* in the sewage group relative to the natural freshwater group. Moreover, the sewage group was predominantly composed of Gram-negative bacteria, with *Escherichia coli* being the most abundant, whereas *Streptococcus pneumoniae* was the most common Gram-positive bacterium. The primary imaging manifestations included exudative and consolidative shading (10). Xiong Limei summarized the results of sputum culture from 164 drowning children and identified *Escherichia coli*, *Streptococcus pneumoniae*, and *Haemophilus influenzae as* the primary pathogens (11). Although sewage inhalation is a critical cause of drowning-related aspiration pneumonia, the reports on *T. whipplei*-positive sputum culture are still lacking. This might be because of the biological characteristics of *T. whipplei*, such as its long culture cycle and susceptibility to contamination. In 2000, Professor Raoult D successfully isolated *T. whipplei* from the cardiac valve of a patient with endocarditis (12). Subsequent research validated the widespread occurrence of *T. whipplei* in European soil and sewage, with elevated detection rates in the saliva and feces of sewer workers relative to normal controls (13, 14). Therefore, individuals with extended soil exposure are at a high risk of infection (15); however, colonization may occur in a latent state without causing acute pneumonia. The initial *T. whipplei* exposure can cause acute bacteremia and pneumonia due to compromised immune function (16). After the 2004 Indian Ocean tsunami, various necrotizing pneumonias and lung abscesses were reported, which were later termed “tsunami lung.” It was closely related to the passive inhalation of large amounts of sediment and seawater by drowning victims (17). The primary imaging characteristics include frosted glass-like opacity patterns and solid lesions. Here, case 1 indicated bilateral ground-glass opacities, which closely resembled those seen in cryptococcal, fungal, or viral infections and may have complicated the diagnosis. Upon admission, blood tests were performed to screen for these potential conditions. Ultimately, *T. whipplei* infection was confirmed through bronchoscopy and mNGS. In case 2, the patient had bilateral pulmonary flocculent lesions, high-density infections, minimal left-sided pleural effusion, and no obvious bronchopneumonia. The left lower lobe of the patient was primarily affected, probably due to the patient’s aspiration position. The patient was first assumed to have co-infections with Gram-negative bacteria and fungi, as indicated by their medical history and imaging results. However, a second-day chest CT assessment indicated a minor reduction in bilateral lung infections. Despite being admitted to the ICU, the patient evaded tracheal intubation and maintained the capacity to spontaneously expectorate sputum, with coughs yielding black, mud-like particles that expedited the absorption of lung infections. In *T. whipplei*-positive patients, nodular and interstitial changes are characteristic imaging features, along with spot and mass shadows, as well as pleural effusions (18). Imaging findings of both cases presented in the study were consistent with previously reported *T. whipplei* pneumonia representations.

The recent advancements in mNGS have increased the rate of *T. whipplei* diagnosis (19). This study also used mNGS to diagnose *T. whipplei* infection, highlighting the advantages of NGS technology in diagnosing complex lung infections. Currently, there are no standard guidelines for treating *T. whipplei* infection (20). Here, the patient in case 1 was initially administered moxifloxacin empirically to inhibit common community-acquired pathogens. After the re-examination, the regimen was then adjusted to CTX (2 g IV) once daily and SMZ twice daily. Subsequently, the patient’s cough and sputum symptoms significantly improved, with no fever observed, confirming the efficacy of the above drug regimen. However, the patient requested immediate discharge without additional imaging follow-up, which complicates confirmation of pulmonary infection absorption, but clinical treatment imaging is not the only criterion for assessing therapeutic efficacy. The efficacy of a treatment is evidenced by the alleviation of patients’ symptoms, such as coughing, expectoration, chest tightness, and dyspnea, with the improvement in their overall quality of life. In case 2, the patient was initially treated with cefoperazone–sulbactam (3.0 IVVP every 8 h) + FLZ (0.4 g IV every day) to prevent Gram-negative bacteria and fungi infection. Currently, carbapenems are used for treating *T. whipplei*; however, in a few cases, patients are first treated with cefoperazone–sulbactam, followed by carbapenems. According to some studies, IV treatment with CTX and Mpm, as well as oral treatment with SMZ and doxycycline, are more effective options (21), but there is significant treatment failure due to drug resistance, and comprehensive antimicrobial course guidelines are lacking. Short-term treatments have reported complete remissions, although some have encountered recurrences following the discontinuation of oral medicine. Unfortunately, there is a lack of large-sample data assessing the timings for symptom and imaging improvement (22). Pulmonary imaging analysis revealed a gradual alleviation of the *T. whipplei* infection; however, the patient still experienced intermittent fever. Furthermore, her CRP levels progressively increased, and neutrophil counts remained consistently above average, indicating that the infection was still active. The subsequent mNGS analysis suggests that the *Aspergillus* infection has not been adequately controlled. Currently, there is no unified consensus on the duration of antibiotic treatment. The symptoms of gas aspiration are often minor, marked by faint imaging findings and few signs of infection. In case 2, symptoms were quickly mitigated after brief antibiotic treatment, and no fever recurrence was observed. However, after an accidental inhalation of sewage, the patient developed systemic infection characterized by persistent fever and increased infection indicators. Moreover, *Aspergillus* infection was also identified. The administration of antibacterial agents improved clinical symptoms and laboratory test results, but intermittent low-grade fevers persisted, indicating a continued need for anti-infection therapy. Unfortunately, the patient’s refusal to continue treatment precluded the completion of the anti-infection medication regimen.

Fungi are a common cause of drowning-related aspiration pneumonia (23). In case 2, a small amount of *Aspergillus flavus* (*Aspergillus* genus) was detected in the lungs. This fungus rarely infects persons with normal immune function but rapidly infects those exposed to fungal environments, particularly patients with impaired immune systems, such as those with AIDS or organ transplant recipients (24). The low mNGS sequence counts suggest that bacterial loads may be inadequate or that the robust cell wall and capsule of *Aspergillus* may impede nucleic acid extraction with mNGS (25). Considering the patient’s absence of immunosuppressive history and drowning exposure, the discovery of aflatoxin was likely due to inhalation during drowning, which also supports the plausibility that *T. whipplei* was inhaled. Voriconazole, posaconazole, and Isavuconazole are effective against *Aspergillus*, whereas FLZ lacks antifungal activity (26). FLZ was initially administered when the patient was in the ICU, probably because of the lack of precise etiological examination results or due to the limited professional knowledge among the treating respiratory physicians. This issue was effectively addressed upon transferring the patient to the respiratory department. The patient’s late-stage imaging results contradict ineffective FLZ treatment for *Aspergillus*, confirming *T. whipplei* as the primary cause of the pulmonary infection. However, muddy water inhalation during drowning can induce fungal infections with severe pneumonia in immunocompetent patients. Symptoms significantly improved after 2 weeks of IV Amphotericin B administration (27). Therefore, voriconazole tablets were orally administered immediately, with an initial dose of 400 mg every 12 h to inhibit *Aspergillus*. The temperature trend chart showed that the patient intermittently experienced fever during hospitalization, with a temperature of 39 °C recorded on September 5. However, voriconazole treatment returned the body temperature gradually to normal. The patient’s PCT peaked on the second day of admission and exhibited a consistent decline. Moreover, white blood cell counts also declined, but the neutrophil count remained at 90% for an extended duration. CRP values reduced before 30th August, followed by a fluctuating increase until reaching a peak of 159 mg/mL on September 4. This might be associated with elevated CRP and neutrophil levels in *Aspergillus* lung infections compared to healthy individuals (28). A telephone follow-up was conducted after the patient was discharged from the hospital. The patient continued to experience sporadic low-grade fever, although the cough symptoms showed considerable improvement. The patient was advised to return to the outpatient department for a follow-up but declined owing to financial constraints regarding hospitalization costs.

## 3 Summary

The shortcomings in managing both cases were poor treatment protocols, inappropriate antibiotic durations, and insufficient imaging follow-ups, typically limited by patient compliance and outside the control of healthcare providers. Upon receiving the mNGS results, the patient started appropriate medication, which improved respiratory symptoms. The patient refused essential diagnostic procedures, while the medical team did not perform gastroscopy and colonoscopy in the initial treatment phase due to insufficient previous experience with analogous cases, delaying the timely verification of the respiratory pathology. The current diagnostic methods for *T. whipplei* infection include PCR, mNGS, and other examination techniques. Considering patients’ finanicial constraints, it is atypical to reaffirm test results with high sequence numbers. If a patient’s treatment response is suboptimal during therapy, it is advised to perform a PCR examination. Histopathological assessment involves invasive procedures such as lung puncture. In cases where therapeutic effects are favorable, histopathological examinations are not performed. Moreover, the accuracy of bacterial detection in saliva samples is inherently lower compared to that of alveolar lavage fluid. In case 1, the patient inhaled an irritant gas of indeterminate chemical composition, potentially compromising future therapy alternatives. In case 2, the absence of a prompt tracheoscopy delayed the collection of etiological results. A misdiagnosis could have significantly exacerbated the patient’s lung infection. Furthermore, the preliminary application of FLZ lacked adequate evidence. Inhaling various substances can lead to significantly diverse disease pathogenesis. In this study, one patient was exposed to gas aspiration, while the other experienced liquid aspiration, providing novel data to improve the current understanding of *T. whipplei* pneumonia.

In the future, cases with clinical pneumonia due to gas and liquid aspiration should be examined for *T. whipplei* and *Aspergillus.* Although *T. whipplei* pneumonia has specific symptoms, imaging features, and signs, it lacks specificity, necessitating precise etiological results. Tracheoscopy and mNGS are essential tools for respiratory physicians to identify complex lung etiologies. Moreover, mNGS can help diagnose more complex lung infections. Although there are limitations in the documented cases, the new findings on the disease etiology, diagnosis, and therapy are of significant value.

## Data Availability

The original contributions presented in the study are included in the article/Supplementary material, further inquiries can be directed to the corresponding author.
