# Supplementary figures and images for: Case Report: Whipple’s disease pneumonia caused by inhalation of gas or liquid
Source: Front Med (Lausanne). 2025 Oct 9;12:1664867. doi: 10.3389/fmed.2025.1664867 (PMC12545080; doi:10.3389/fmed.2025.1664867)

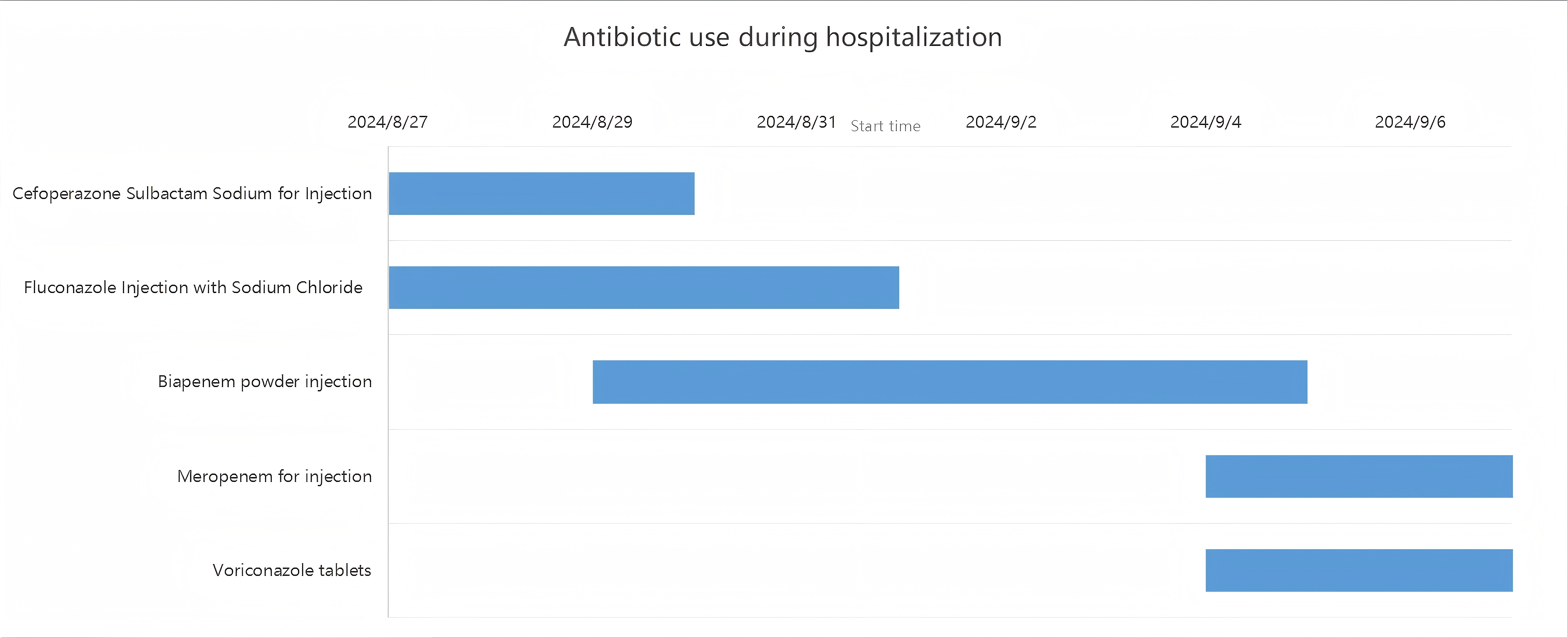

Supplement: Supplementary file 1 [file Image_1.jpeg]
